# Antibacterial substantivity of Carvacrol and sodium hypochlorite in infected bovine root dentin

**Published:** 2009-04-17

**Authors:** Mohammad Reza Sharifian, Noushin Shokouhinejad, Marzieh Aligholi, Mohammad Emaneini, Javad Alizadeh

**Affiliations:** 1 *Department of Endodontics, Faculty of Dentistry/Dental Research Center, Tehran University of Medical Sciences, and member of Iranian Center for Endodontic Research, Tehran, Iran.*; 2 *Instructor of Microbiology, School of Medicine, Tehran University of Medical Sciences, Tehran, Iran.*; 3 *Department of Microbiology, School of Medicine, Tehran University of Medical Sciences, Tehran, Iran.*; 4 *Postgraduate Student of Endodontics, Faculty of Dentistry, Tehran University of Medical Sciences, Tehran, Iran.*

**Keywords:** Agents, Antimicrobial, Carvacrol, Sodium hypochlorite, Substantivity

## Abstract

**INTRODUCTION:** Various methods commonly used for cleaning and shaping root canals have not been successful in completely eradicating bacteria due to anatomic complexity and root canals irregularities. Disinfecting the canals with intracanal irrigants in addition to proper cleaning and shaping can produce a successful outcome. Antimicrobials with sustained antibacterial activity would be desirable for irrigation. The purpose of this study was to compare the antimicrobial substantivity of Carvacrol and 5.25% NaOCl in infected bovine root dentin.

**MATERIALS AND METHODS:** One hundred and twenty dentin tubes prepared from bovine incisors were infected *in vitro* for 14 days with *Enterococcus (E) faecalis*. The specimens were divided into four groups including 1) Carvacrol, 2) NaOCl, 3) infected dentin tubes (positive control); and 4) sterile dentin tubes (negative control). Dentin chips were collected at five intervals (days 0, 1, 3, 7 and 28) using round burs with sequentially increasing diameters (which includes five layers of dentin) into Brain Heart Infusion (BHI) broth. In order to compare the pre- and post-irrigation antimicrobial activity of the irrigants the colony-forming units (CFU) were counted and classified as ‘CFU-before’ and ‘CFU-after’. After culturing, the number of CFU with the various experimental time and dentinal layers was recounted. Two-way ANOVA test was used to analyze the effects of time and materials. One-way ANOVA and supplemental Tukey HSD test were used for pair comparison.

**RESULTS:** CFU was significantly reduced in NaOCl group when compared to all other experimental groups (P<0.05).

**CONCLUSION:** The substantivity of NaOCl was significantly greater than Carvacrol. Further studies are required to investigate and approve Carvacrol as a final irrigant.

## INTRODUCTION

Bacteria that remain or re-colonize in a obturated root canal system are the chief cause of endodontic failure (1,2). The bacteria in the root canal system not only invade the anatomic irregularities of the root canal system but also penetrate into dentinal tubules (3). Numerous measures have been described to reduce the numbers of root canal bacteria, including various instrumentation techniques, irrigation regimens and intracanal medicaments (4). An ideal intracanal irrigant or medicament should be able to disinfect the dentin and its tubules in one visit. In addition, it should have sustained antimicrobial effect after use. Furthermore, it must be biocompatible with live host tissue (3).

The most commonly used proteolytic material is NaOCl (5). NaOCl dissolves necrotic tissues and debris through a complex biochemical process. Concentration of free chlorine is important for this breakdown of proteins into amino acid groups (6). Increased temperature also potentiates the antimicrobial and organic tissue catabolism of NaOCl (7).

Carvacrol (2-methyl-5-isopropylphenol) is a combination of plant essential oils such as oregano (origanum vulgare), thyme (thymus vulgaris), and savory (satureja hortensis) (8). It has well-known antibacterial and antifungal properties as well as antioxidative effects, with a number of potential applications for topical treatment of mucosal and epithelial infection (9). Carvacrol is able to disintegrate the outer membrane of gram negative bacteria, release lipopolysacharides and increase the permea-bility of cytoplasmic membrane to ATP (10).

Studies regarding NaOCl antibacterial substantivity have been controversial. Khademi *et al.* demonstrated shorter substantivity for NaOCl, compared to Doxycycline and chlorhexidine at days 0, 1, 3, 7 and 28 (11), whilst Mohammadi *et al.* (12) found no substantivity for NaOCl at days 0, 7, 14 and 21.

Carvacrol's antibacterial substantivity has not been yet evaluated. The purpose of this study was to compare the antibacterial substantivity of Carvacrol and NaOCl against *Enterococcus *(*E*)* faecalis* in bovine root dentin.

## MATERIALS AND METHODS

Fifty bovine incisors were selected for this study. The specimens were kept in 0.5% NaOCl solution for seven days or less.

The apical 5 mm and two-thirds of the crown were removed from each tooth with a rotary diamond saw (Isomet Plus precisan saw, Buchler, IL, USA) at 1,000 rpm with water coolant. The roots were then cut into 4 mm-thick slices with the diamond saw. The canals were enlarged using low speed ISO 023 round bur. To avoid dehydration teeth and the blocks were preserved in containers of tap water. All blocks were mounted in self-cure acryl. One end of the blocks was sealed with light cure glass ionomer for preventing the acrylic hernia into the blocks. The dentin tubes were individually treated with 17% EDTA for one minute and then with 5.25% NaOCl to remove the smear layer. The specimens were autoclaved. They were then kept in an incubator at 37^°^C for 24 hours to check the efficacy of the sterilization.

Five specimens were randomly selected. Dentin chips were removed from the canals with sequential sterile low speed round burs with increasing diameter, and then the powdered dentin samples obtained with each bur were immediately collected and cultured on blood agar. This step was performed to confirm that complete sterilization had taken place.

Isolated 24-hour colonies of pure cultures of *E. faecalis* (ATCC 29212) were suspended in Brain Heart Infusion (BHI). They were inoculated into the canals and then the specimens were kept at 37^°^C for 14 days with a replacement of 1 mL of freshly prepared BHI every 2 days. In the negative control group sterile BHI was replaced every 2 days.

Subsequent to the contamination period (before irrigating the specimens), the very first samples were obtained from within the whole canals and were recorded as the first number of colony forming units (CFU-1).

Also five randomly selected specimens were enlarged with sequential sterile low speed round burs with increasing diameters of ISO sizes 025, 027, 029, 031, and 033. Each bur removed approximately 0.1 mm of root canal dentin. Removed dentin layers were named according to the burs diameter hence the first layer would be removed by bur ISO 025 and the fifth Layer would be removed by bur ISO 033. After recording CFU-1, a total 120 specimens (dentin tubes) were randomly divided into four groups as follows: Group 1 (n=50); NaOCl 5.25% (n=10) (Sigma Chemical Co., MO, USA), were tested at each experimental time of 0, 1, 3, 7 and 28 days. Group 2 (n=50) consisted of Carvacrol 10% (Khoraman Co.), again with 10 specimens for each time variable. Group 3 (n=10) consisted of positive control (infected dentin tubes) with 2 specimens for each time variable.

Dentin tubes in group 1 and 2 were filled with the test solutions. In group 1, NaOCl was inserted for 20 min and in group 2, Carvacrol was inserted for 20 min. Then the canals were dried by paperpoints (Aryadent, Tehran, Iran) and irrigated with normal saline. The CFU-2 was recorded after this step in order to determine the diminution of the bacteria from the canals. The specimens were then incubated at 37^°^C for 28 days.

**Table 1:** Means±SD of the reduced CFU (percentage) of *E. faecalis* in experimental groups

**Groups**	**CFU reduction (%) ± SD**				
	**Day 0**	**Day 1**	**Day 3**	**Day 7**	**Day 28**
**Carvacrol**	91.69 ± 3.22	91.53 ± 8.66	90.1 ± 9.43	95.46 ± 5.99	99.3 ± 1.54
**NaOCl**	94.97 ± 9.18	97.09 ± 17.07	99.99 ± 0.004	98.17 ± 4.54	99.98 ± 0.04
**Positive control**	65.75 ± 18.03	65.12 ± 5.22	77.25 ± 2.52	80.70 ± 12.07	83.23 ± 7.31

During this period, the allotted specimen for any given time of 0, 1, 3, 7 and 28, were enlarged with the sequentially increasing round burs (with diameters of ISO sizes 025, 027, 029, 031 and 033. Each bur removed approximately 0.1 mm of dentin around the canal. The powdered dentin samples obtained with each bur were collected in separate test tubes containing 3 mL of freshly prepared BHI. After incubation at 37^°^C for 48 hours, the tubes were evaluated regarding the turbidity of microbial growth, and the samples with microbial growth were recorded as positive. In rule out the cross-contamination samples obtained from the turbid tubes were cultured on BHI Agar under aerobic condition. Then gram staining and microbial identification tests were carried out. Growing colonies were counted and recorded as CFU.

Two-way ANOVA was performed to analyze the effect of time and material on bacterial reduction. Since the interactions of variables were meaningful, One-way ANOVA test and Tukey test were used to pair comparison.

## RESULTS

With regards to the reduced CFU, one-way ANOVA test showed significant difference between test materials at days 0, 1, 3, 7, and 28 (P<0.05). The positive control group showed viable bacteria at all experimental times. In contrast the negative group displayed no bacteria at all time intervals. NaOCl group demonstrated the most efficient antibacterial action (P<0.05) at all experimental times (Table 1). Also, the substantivity of NaOCl group was significantly greater than Carvacrol at all time intervals; being present in root dentin for at least 28 days.

## DISCUSSION

*Enterococci *are gram-positive cocci that exist as single units, in pairs, or as short chains. They are facultative anaerobes, possessing the ability to grow in the presence or absence of oxygen (13). *E. faecalis* is a normal inhabitant of the oral cavity (14). It has a higher prevalence in oral rinse samples from patients receiving endodontic treatment and retreatment compared to those with no endodontic history (15*). E. faecalis* is found in 4-40% of primary endodontic infections (16). The prevalence of *E. faecalis* in root-filled teeth with periradicular lesions using cultures and polymerase chain reaction (PCR) is 24-70% and 67-77%, respectively (16)*. E. faecalis* possesses several virulence factors which contribute to its pathogenicity; moreover, its ability to survive and persist as a pathogen in the root canals provides the greatest advantage (14). *E. faecalis* was selected because this bacterium has been identified as one of the most difficult bacterial species to eradicate from the root canal.

The rationale for selecting bovine dentin blocks was its appropriate thickness for removing dentin chips with the sequential enlarging burs. Moreover, separate dentin powder from each layer was collected to confirm the bacterial growth into the whole dentinal depth to verify the negative culture test after using irrigants.

To date, numerous studies have evaluated the antibacterial activity of Carvacrol against bacterial species. Nostro *et al. *showed the antibacterial effect of Carvacrol against methicilin-resistant and methicilin-sensitive *staphylococci *(13). Knowels *et al.* described the antibacterial activity of Carvacrol against *staphylococcus* and *salmonella *(14). Gill *et al.* implied that Carvacrol is able to disintegrate the cellular membrane of bacteria, increasing the non-specific permeability of cytoplasmic membrane and stopping ATPase activity (15).

Sharifian *et al.* showed that 0.3% Carvacrol was bacteriostatic against *E. faecalis* and 0.6% Carvacrol removed *E. faecalis* completely from the culture (16). They found that there was no difference in antimicrobial efficacy for irrigation with 0.6% Carvacrol versus 5.25% NaOCl.

Regarding residual antibacterial activity, Khademi *et al.* compared the antibacterial substantivity of 2% CHX, 100mg/mL Doxycy-cline, and 2.6 %NaOCl in bovine root dention *in vitro* (9). They found that substantivity of CHX was significantly greater than Doxycy-cline and NaOCl, conflicting with Mohammadi *et al.’s* results (12). They compared the antimicrobial substantivity of MTAD, 2% CHX and 2.6% NaOCl in human root dentin *in vitro*. Their findings showed that the substantivity of MTAD was significantly higher than CHX and was sustained in root canal dentin for at least 28 days. Furthermore, NaOCl displayed no substantivity in contrast to the present study. This study showed that when comparing 5.25% NaOCl versus Carvacrol there was no difference in the CFU count before and after irrigation. Another significant observation in the our study was that the substantivity of 5.25% NaOCl was significantly greater than Carvacrol at all time intervals and retained in root canal dentin for at least 28 days.

This variation in results may be due to the fact that we obtained dentin samples from all the sequentially increasing diameters of the root canals at the various times and the residual antibacterial activity within dentinal tubules.

Also, the results of dentin chip cultures in the individual samples were based on a binary code, meaning that if the first layer of the dentin chips showed growing colonies the result would be recorded as zero, and it was recorded as one if growing colony was not observed. Then the numerical result would be added and statically analyzed.

Finally, in previous studies CFU count was not recorded before and after irrigation. The rationale of this record is to confirm the contamination period and also to verify the antimicrobial activity of the irrigants.

## CONCLUSION

In conclusion, we found that the substantivity of sodium hypochlorite was significantly greater than Carvacrol. Further studies are required to investigate these results.

## References

[1] Sjögren U, Figdor D, Persson S, Sundqvist G (1997). Influence of infection at the time of root filling on the outcome of endodontic treatment of teeth with apical periodontitis. Int Endod J.

[2] Molander A, Reit C, Dahlén G, Kvist T (1998). Microbiological status of root-filled teeth with apical periodontitis. Int Endod J.

[3] Torabinejad M, Handysides R, Khademi AA, Bakland LK (2002). Clinical implications of the smear layer in endodontics: a review. Oral Surg Oral Med Oral Pathol Oral Radiol Endod.

[4] Rosenthal S, Spångberg L, Safavi K (2004). Chlorhexidine substantivity in root canal dentin. Oral Surg Oral Med Oral Pathol Oral Radiol Endod.

[5] Bystrom A, Sundqvist G (1985). The antibacterial action of sodium hypochlorite and EDTA in 60 cases of endodontic therapy. Int Endod J.

[6] Harrison JW (1984). Irrigation of the root canal system. Dent Clin North Am.

[7] Cunningham WT, Balekjian AY (1980). Effect of temperature on collagen-dissolving ability of sodium hypochlorite endodontic irrigant. Oral Surg Oral Med Oral Pathol.

[8] Andersen A (2006). Final report on the safety assessment of sodium p-chloro-m-cresol, p-chloro-m-cresol, chlorothymol, mixed cresols, m-cresol, o-cresol, p-cresol, isopropyl cresols, thymol, o-cymen-5-ol, and carvacrol. Int J Toxicol.

[9] Chami N, Chami F, Bennis S, Trouillas J, Remmal A (2004). Antifungal treatment with carvacrol and eugenol of oral candidiasis in immunosuppressed rats. Braz J Infect Dis.

[10] Helander IM, Alakomi HL, Latva-Kala K, Mattila-Sandholm T, Pol I, Smid EJ, Gorris LGM, von Wright A (1998). Characterization of the action of selected essential oil components on Gram-negative bacteria. Journal of Agricultural and Food Chemistry.

[11] Khademi AA, Mohammadi Z, Havaee A (2006). Evaluation of the antibacterial substantivity of several intra-canal agents. Aust Endod J.

[12] Mohammadi Z, Shahriari S (2008). Residual antibacterial activity of chlorhexidine and MTAD in human root dentin in vitro. J Oral Sci.

[13] Nostro A, Blanco AR, Cannatelli MA, Enea V, Flamini G, Morelli I, Sudano Roccaro A, Alonzo V (2004). Susceptibility of methicillin-resistant staphylococci to oregano essential oil, carvacrol and thymol. FEMS Microbiol Lett.

[14] Knowles JR, Roller S, Murray DB, Naidu AS (2005). Antimicrobial action of carvacrol at different stages of dual-species biofilm development by Staphylococcus aureus and Salmonella enterica serovar Typhimurium. Appl Environ Microbiol.

[15] Gill AO, Holley RA (2006). Disruption of Escherichia coli, Listeria monocytogenes and Lactobacillus sakei cellular membranes by plant oil aromatics. Int J Food Microbiol.

[16] Sharifian MR, Nosrat A, Aligholi M An in vitro evaluation of the antimicrobial effect of carvacrol on Enterococcus faecalis as final intra canal irrigant and dressing.

